# Life table and consumption capacity of corn earworm, *Helicoverpa armigera* , fed asparagus, *Asparagus officinalis*

**DOI:** 10.1093/jis/14.1.34

**Published:** 2014-01-01

**Authors:** Ratna Kumar Jha, Shu-Jen Tuan, Hsin Chi, Li-Cheng Tang

**Affiliations:** 1 Department of Entomology, National Chung Hsing University, Taichung 402, Taiwan, Republic of China; 2 Plant Protection Directorate, Department of Agriculture, Hariharbhawan, Lalitpur, Nepal

**Keywords:** intrinsic rate of increase, net consumption rate, resampling

## Abstract

The life table and consumption rate of *Helicoverpa armigera* (Hübner) (Lepidoptera: Noctuidae) reared on asparagus, *Asparagus officinalis* L. (Asparagales: Asparagaceae) were studied under laboratory conditions to assess their interaction. Development, survival, fecundity, and consumption data were analyzed by the age-stage, twosex life table. This study indicated that asparagus is a natural host of *H. armigera.* However, the poor nutritional content in asparagus foliage and the poor fitness of *H. armigera* that fed on asparagus indicated that asparagus is a suboptimal host in comparison to hybrid sweet corn. The uncertainty associated with life table parameters was estimated by using jackknife and bootstrap techniques, and the results were compared for statistical inference. The intrinsic rate of increase ( *r* ), finite rate of increase ( *λ* ), net reproductive rate ( *R* 0), and mean generation time ( *T* ) were estimated by the jackknife technique to be 0.0780 day ^-1^ , 1.0811 day ^-1^ , 67.4 offspring, and 54.8 days, respectively, while those estimated by the bootstrap technique were 0.0752 day ^-1^ , 1.0781 day ^-1^ , 68.0 offspring, and 55.3 days, respectively. The net consumption rate of *H. armigera* , as estimated by the jackknife and bootstrap technique, was 1183.02 and 1132.9 mg per individual, respectively. The frequency distribution of sample means obtained by the jackknife technique failed the normality test, while the bootstrap results fit the normal distribution well. By contrast, the relationship between the mean fecundity and the net reproductive rate, as estimated by the bootstrap technique, was slightly inconsistent with the relationship found by mathematical proof. The application of the jackknife and bootstrap techniques in estimating population parameters requires further examination.

## Introduction

*Helicoverpa armigera* (Hübner) (Lepidoptera: Noctuidae) is a widely distributed insect pest of high agricultural importance and is listed as a quarantine pest by the European and Mediterranean Plant Protection Organization ( EPPO 2008 ). *H. armigera* larvae feed on foliage or on the reproductive organs of plants and usually cause substantial economic losses ( Reed and Pawar 1982 ). A wide range of field crops, horticultural crops, and floricultural plants are recorded as host species of *H. armigera* ( Zalucki et al. 1986 , 1994). *H. armigera* has also been reported in asparagus fields in Taiwan and Australia ( COA 1996 ; Bussell et al. 2002 ; Fei et al. 2010 ). No information, however, is currently available on the life table and consumption capacity of *H. armigera* on asparagus.

Asparagus, *Asparagus officinalis* L. (Asparagales: Asparagaceae) is an herbaceous perennial plant with erect stems and branched, feathery foliage consisting of small, green, needle-like structures called cladodes. Cladodes are modified stems in the axils of scale leaves. Asparagus plants have no functional leaves. The tender shoots, commonly known as spears, are eaten as a vegetable. Although it is grown in temperate zones, it has been developed into a surprisingly successful industry in Taiwan, a subtropical and tropical area ( Hung 1980 ). This vegetable is of great importance in diets because of its high nutrient content; it contains valuable salts and vitamins, is high in cellulose, and also has multipurpose therapeutic properties.

Characterization of the growth, stage structure, fecundity, and consumption rate of an insect pest is essential to understand its interactions with host plants and physical environments. Characterization can be facilitated by proper analyses of life history data and daily consumption via life-table theory. Life-table theory is used in diverse fields related to population ecology ( Wilcox and Murphy 1985 ; Chi and Getz 1988 ; Chi 1990 ; Chi 1994 ; Sakai et al. 2001 ; Stark and Banks 2003 ; Stark et al. 2007 ). A life table provides the most comprehensive description of a cohort of individuals or of a typical individual from a given population in terms of survival, development, and reproduction ( Price 1997 ; Yu et al. 2005 ; Yang and Chi 2006 ). The intrinsic rate of increase is the most useful and appropriate life-table parameter for comparing the fitness of populations across diverse climatic and food-related conditions ( Southwood 1966 ; Smith 1991 ). Similarly, the net consumption rate represents the consumption or predation capacity of an insect population, including all individuals of both sexes and those that died before reaching the adult stage ( Chi and Yang 2003 ; Farhadi et al. 2011 ). These indicators are important to comprehend the performance of an herbivore as well as that of its natural enemies in the ecosystem.

In this study, the age-and stage-specific consumption rates were integrated into the age-stage, twosex life table of *H. armigera* reared on asparagus. Then the demographic characteristics of this population were estimated by using the age-stage, twosex life table and compared with characteristics of a population reared on hybrid sweet corn. The application of the jackknife and bootstrap techniques in estimating standard errors of population parameters are also discussed. This study demonstrates the advantages of incorporating consumption data into the age-stage, twosex life table.

## Materials and Methods

### Asparagus

Asparagus foliage was obtained from plants grown at Caotun County in a field without pesticides. During the experimental period (October to December 2010), weeds were removed by hand. A batch of healthy young foliage was brought from the field every two to three days during the experiment. The lateral branches were excised, and the stem portion of the excised branches was dipped into water to protect the foliage from drying. The foliage was provided to *H. armigera* as food.

### H. armigera

The founding colony of this pest was collected from fields in Taichung County and maintained in the Microbial Control Laboratory, Department of Entomology, National Chung Hsing University Taichung, Taiwan (R.O.C). The colony was periodically supplemented with larvae collected from the field to reduce inbreeding depression. Before the study began, experimental insects were reared on asparagus for one generation in a growth chamber at 25 ± 1°C and 65 ± 5 % RH, with a photoperiod of 14:10 L:D.

### Life table and consumption rate study

Newly-emerged adults from the laboratory colony were paired and kept in individual oviposition containers (plastic cup 9 cm in diameter and 5.5 cm in height, lined with paper towels). The adults were provided daily with a cotton ball soaked with 30% honey solution. Eggs from 12 females laid on the same day were collected in Petri dishes (9 cm diameter) and kept separately in the growth chamber at 25 ± 1°C and 65 ± 5 % RH, with a photoperiod of 14:10 L:D. The egg hatch rates were observed daily. A total of 110 newly-hatched larvae were individually transferred to Petri dishes (9 cm diameter) with a fine brush and reared in groups up to the 2 ^nd^ instar. The 3 ^rd^ and older instars were reared individually in similar Petri dishes. Individual larvae were observed daily for molting and survivorship. Weighed fresh asparagus foliage was provided to larvae daily by wrapping the bottom (cut-end) of the stem with moist cotton to protect the foliage from rapid desiccation. The water loss from the asparagus foliage was estimated from the similar foliage (control) kept without larvae in a container similar to those used for rearing. This wrapping was removed before the final weighing, after 24 hr. The amount of asparagus foliage consumed by a larva within 24 hr was corrected for water loss and calculated as follows (Waldbaure 1968):

Amount of foliage consumed =

$(1 - \frac{\alpha}{2})[I - (F + \beta F)]$

where *I* is the initial weight of foliage introduced as food and *F* is the weight of uneaten foliage after 24 hr. Thus,

$\alpha = w/I_c\, \text{and}\, \beta = w/F_c$

where *Ic* is the initial weight of foliage of the control treatment (without insect), *Fc* is the final weight of foliage of the control treatment after 24 hr, and *w* is amount of water loss; *w* = *Ic* – *Fc* .

Larvae entering the prepupal stage were provided with decomposed-peat-based compost (blocking compost by Plantaflor Humus Verkaufs GmbH, D 49377, www.plantaflor.de ) for pupation. Each pupa was sexed, weighed, and then kept individually in plastic cups (9 cm diameter and 5.5 cm height). Newly-emerged adults were paired in oviposition containers lined with paper towels. The adults were transferred to a new container, and eggs were collected daily. Eggs laid by each female at different ages were kept separately to record the hatch rates. The entire study was carried out under the same conditions as those prior to the experiment in the growth chamber.

### Foliar chemical analyses

Water, nitrogen, and total nonstructural carbohydrates contents were quantified for asparagus foliage. Similar foliage used for feeding insects for the consumption study were cut from nine randomly selected plants, placed separately in paper envelopes, kept inside a plastic bag on ice, and carried immediately to the laboratory. For quantifying water content, some of asparagus foliage from each sample was weighed for the wet weight, oven dried at 60°C for 1 week, and then reweighed to record dry weight. Percent foliar water content was calculated using the wet and dry weight values of these leaves. For quantifying nitrogen and total nonstructural carbohydrate content, the sample foliage kept in paper envelopes was flash-frozen in liquid nitrogen for 30 min, vacuum-dried for 24 hr, ground, and stored at -20°C for chemical analyses. The nitrogen and total nonstructural carbohydrate contents of each foliage sample were measured by following the procedures as used by Yadav et al. (2010) .

### Life-table analysis

The raw data from the life table were analyzed based on the theory of the age-stage, twosex life table ( Chi and Liu 1985 ; Chi 1988 ). The developmental periods for each development stage of all individuals (including males, females, and those that died before reaching the adult stage), as well as the adult preoviposition period, the total preoviposition period, and female fecundity, were calculated. The adult preoviposition period is calculated based on time after the emergence of an adult female insect, while the total preoviposition period is based on the time since birth and thus represents the “true” figure. The basic life-table parameters, namely the age-stage-specific survival rate ( *sxj* ) (where *x* is the age and *j* is the stage), the age-stage-specific fecundity ( *fxj* ), the age-specific survival rate ( *lx* ), and the age-specific fecundity ( *mx* ), were calculated from daily records of the survival and fecundity of all individuals in the cohort. The age-stage-specific fecundity ( *fxj* ) was calculated from the numbers of hatched eggs, as this parameter reflects the true biological characteristics of *H. armigera* . The population parameters ( *r* , the intrinsic rate of increase; *λ* , the finite rate of increase, *λ* = *e ^r^* ; *R* 0, the net reproductive rate; *T* , the mean generation time) were calculated accordingly. In this paper, the intrinsic rate of increase was estimated by the iterative bisection method from the Euler-Lotka formula (equation 1):

$\sum_{x = 0}^{\infty}e^{-r(x + 1)}l_{x}m_{x} = 1$

with age indexed from 0 ( Goodman 1982 ). The bisection method can be found in most textbooks of numerical analysis ( Burden and Faires 2005 ). The mean generation time ( *T* ) is defined as the period that a population needs to increase to *R* 0-fold its starting size as the stable age-stage distribution and the stable increase rate (i.e., *r* and *λ* ) are reached. In other words:

$e^rT = R_0\, or \lambda^{T} = R_0$

The mean generation time was calculated as:

$T = ln R_0/r$

The gross reproductive rate ( *GRR* ) was calculated as:

$GRR = \sum m_{x}$

Because life-table studies are extremely time-consuming and replication is impractical, the means and standard errors of the life-table parameters were estimated by both the jackknife and bootstrap techniques ( Meyer et al. 1986 ; Sokal and Rohlf 1995 ) included in TWOSEX-MSChart ( Chi 2010 ), and results were compared. The age-stage life expectancy was calculated by following the procedures described in Chi (1988) and Chi and Su (2006) . Because the hatch rate of eggs varied with age, the unhatched eggs were excluded from the parent cohort and the fecundity data. The raw-data analysis and estimation of life-table parameters were performed via a user-friendly computer program, TWOSEX-MSChart ( Chi 2010 ), designed in Visual BASIC (Version 6.0 Service pack 6) for Windows (Microsoft, www.microsoft.com ). The age-stage-specific reproductive value ( *vxj* ) was also calculated using TWOSEX-MSChart. The Mann-Whitney test (U-test) (Sigmaplot 11.0, Systat Software Inc., www.systat.com ) was used to determine the difference in development times, fecundities, and population parameters of *H. armigera* reared on asparagus and hybrid sweet corn by reanalyzing the hybrid sweet corn life table raw data used in Jha et al. (2012) .

### Consumption rate analysis

The daily consumption data for all individuals, including those that died before reaching the adult stage, were used to calculate the age-stage-specific consumption rate ( *cxj* ). The age-stage-specific consumption rate duly considers the stage differentiation and the variable consumption rate among individuals. The *cxj* gives the average amount of asparagus foliage consumed by an individual of *H. armigera* of age *x* and stage *j.* The age-specific consumption rate ( *kx* ), the age-specific net consumption rate ( *qx* ), and the net consumption rate ( *C* 0) were represented as *cxj* . The equation also takes into account the age-stage-specific survival rate ( *sxj* ). The age-specific consumption rate ( *kx* ) is the average amount of asparagus leaf consumed by an individual *H. armigera* of age *x* and is calculated as (equation 2):

$k_{x} = \frac{\sum_{j = 1}^{\beta}S_{xj}C_{xj}}{\sum_{j=1}^{\beta}S_{xj}}$

where *β* is the number of life stages *.* Taking the survival rate into consideration, the age-specific net consumption rate ( *qx* ) gives the weighted amount of asparagus leaf consumed by *H. armigera* larvae of age *x* and is calculated as (equation 3):

$q_{x} = k_{x}l_{x}$

According to Chi and Liu (1985) , the age specific survival rate ( *lx* ) is calculated as (equation 4):

$l_x = \sum_{j = 1}^{\beta}s_{xj}$

The net consumption rate ( *C* 0) is defined as the summation of the *qx* over all age groups, giving (equation 5):

$C_0 = \sum_{x=0}^{\delta}q_{x} = \sum_{x=0}^{\delta}k_{x}l_{x} = \sum_{x=0}^{\delta}\sum_{j = 1}^{\beta}S_{xj}C_{xj}$

where *δ* is the last age of the population. The parameter *C* 0 is the total amount of asparagus leaf consumed by an average individual during its life span. It is a demographic parameter that represents the consumption capacity of a pest population, including all individuals of both sexes and those that died before the adult stage. The transformation rate from food mass to pest offspring ( *Qc* ) is the ratio of the net consumption rate to the net reproductive rate ( Chi and Yang 2003 ) and is calculated as (equation 6):

$Q_c = \frac{C_0}{R_0}$

*Qc* gives the quantity of food needed for the production of an offspring. Consumption rate data were analyzed using the computer program CONSUME-MSChart, as designed by Chi (2012) . The standard errors of the consumption parameters were also calculated using both the jackknife and the bootstrap techniques.

## Results

Out of 110 1 ^st^ instar individuals reared on asparagus foliage, only 54 individuals (49.1%) completed their larval stage and survived to the pupal stage. Among those 54 individuals, 35 (64.8%) completed their larval stage at the 6 ^th^ instar, 17 (31.5%) completed their larval stage at the 7 ^th^ instar, and 2 (3.7%) completed their larval stage at the 5 ^th^ instar ( Table 1 ). The duration of and survival at each stage, as well as adult longevity, preoviposition period, oviposition duration, and female fecundity, of *H. armigera* reared on asparagus and hybrid sweet corn are compared in Table 1 . The results showed that larvae of *H. armigera* reared on asparagus in the laboratory were able to complete their development to the adult stage and produce fertile offspring. The stage-specific durations (days) of *H. armigera* larvae that developed to the adult stage from the 6 ^th^ instar and from the 7 ^th^ instar are compared in Table 2 . Despite the significant differences in the duration of development of 4 ^th^ instar ( *P* = 0.048), 5 ^th^ instar ( *P* = 0.004), and 6 ^th^ instar ( *P* = 0.019) *H. armigera* when compared with those that developed to the adult stage from the 6 ^th^ and 7 ^th^ instars, there was no significant difference in the total larval period ( *P* = 0.934). The probability that a newly-hatched larva would develop to the adult stage from the 6 ^th^ instar (0.1818) was considerably higher than the probability of development from the 7 ^th^ instar (0.1273). The probability of a newly hatched larva surviving to the adult stage was 0.3217, which is considerably less than that reported for larvae fed hybrid sweet corn and artificial diet ( Jha et al. 2012 ).

**Table 1.**
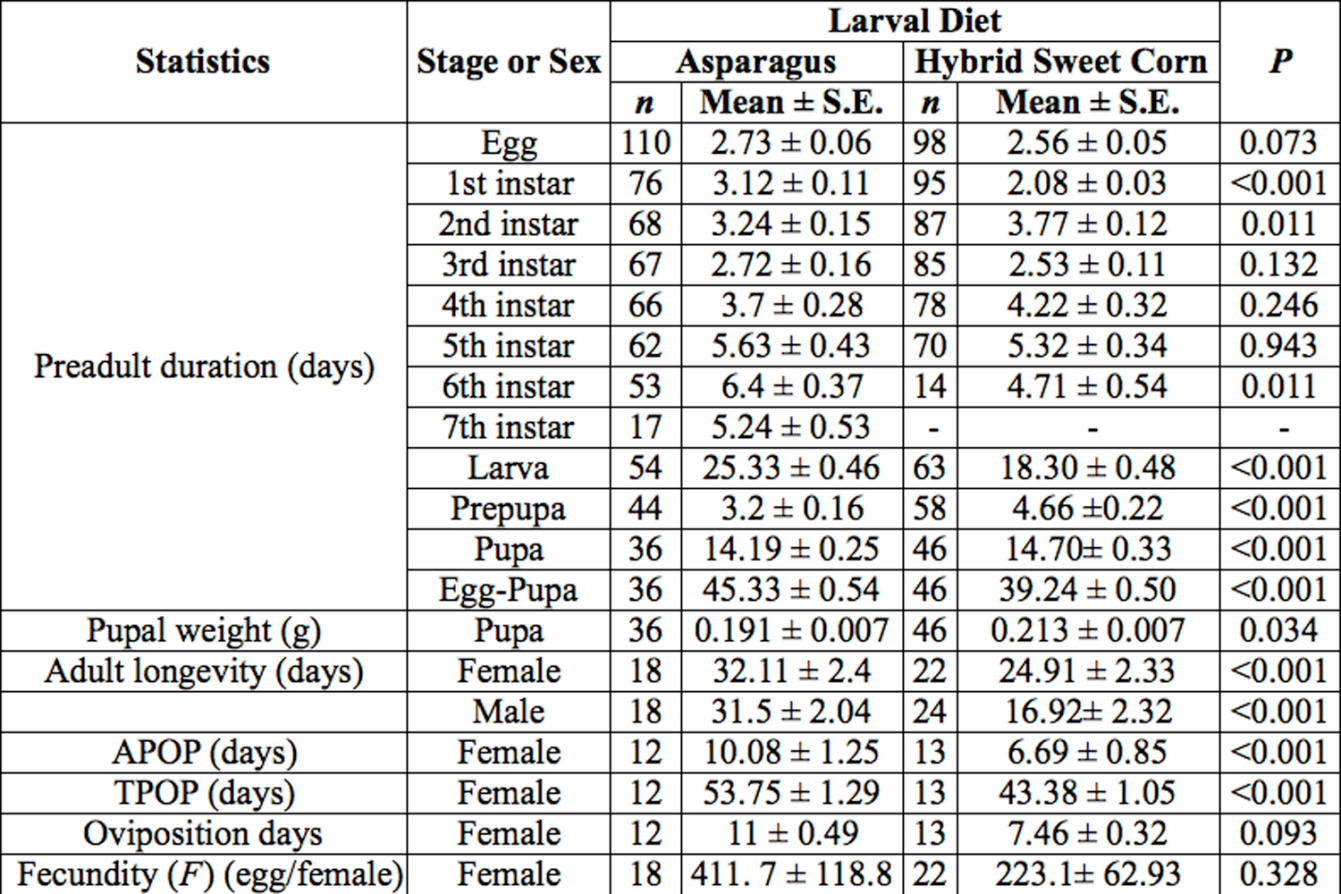
Comparison of basic life history statistics of *Helicoverpa armigera* fed on asparagus and hybrid sweet corn.

All *P* -values are calculated from the U-test except the *P* -values of pupal weight and oviposition days. APOP (adult preoviposition period) and TPOP (total preoviposition period) were calculated using females that produced fertile eggs.

**Table 2.**
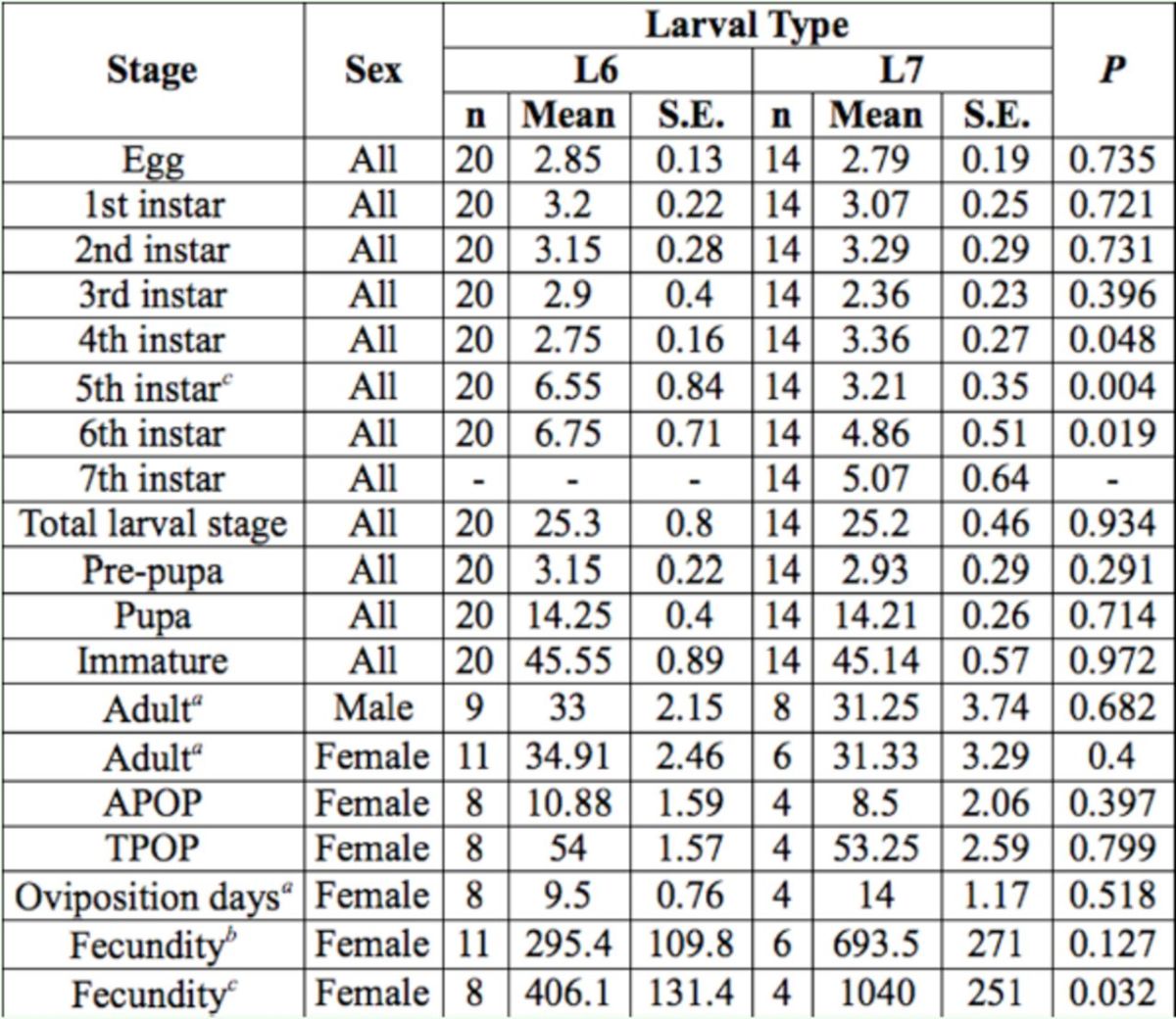
Stage-specific duration (days) of *Helicoverpa armigera* larval development to the adult stage from 6th instar (L6) and 7th instar (L7) larvae reared on asparagus

All *P* -values calculated from U-test. APOP (adult preoviposition period) and TPOP (total preoviposition period) were calculated using females that produced fertile eggs.

*^a^ P* -values of these stages are calculated by the t-test, as the data pass the normality assumption.

*^b^* All female adults are included.

*^c^* Only reproductive female adults are included

The mean fecundity of *H. armigera* reared on asparagus was 411.7 fertile eggs. The mean fecundity of *H. armigera* that developed to the adult stage from the 7 ^th^ instar (639.5 eggs) was considerably higher than that of individuals that developed from the 6 ^th^ instar (295.4 eggs) ( Table 2 ). This difference was statistically insignificant; however, a significant difference was observed when only reproductive females were included in the comparison.

**Figure 1.**
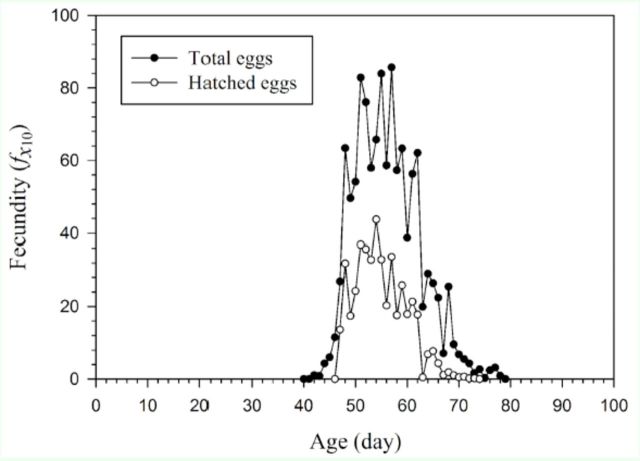
Total number of eggs and hatched eggs laid by female *Helicoverpa armigera* at different ages. High quality figures are available online.

The gap between the curves showing the age-specific total eggs laid and the age-specific eggs hatched in Figure 1 represents the difference between the number of eggs laid and the number that actually hatched. The figure also shows the changes in the age-specific hatch rate with age. This variation has been taken into consideration in the calculation of population parameters.

**Figure 2.**
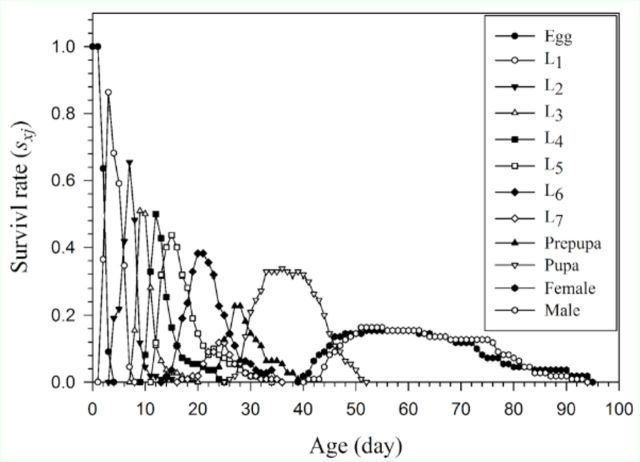
Age-stage-specific survival rate ( *sxj* ) of *Helicoverpa armigera* reared on asparagus. High quality figures are available online.

**Figure 3.**
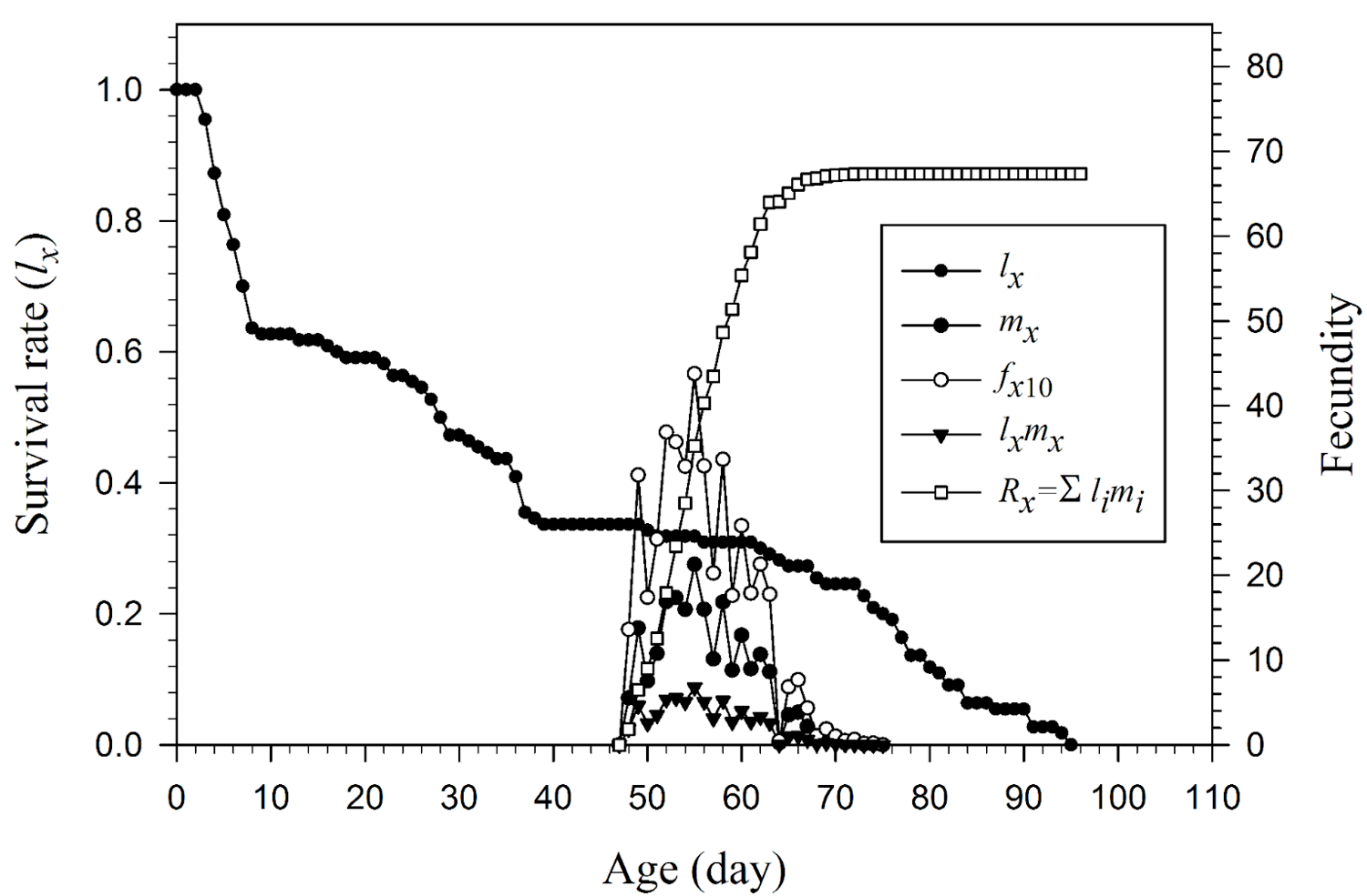
Age-specific survival rate ( *lx* ), female age-specific fecundity ( *fx* 10), age-specific fecundity of the total population ( *mx* ), age-specific maternity ( *lxmx* ), and cumulative reproductive rate ( *Rx* ) of *Helicoverpa armigera* reared on asparagus. High quality figures are available online.

The age-stage, twosex life table revealed the overlap of the age-stage-specific survivorship ( *sxj* ) curve of the cohort of *H. armigera* reared on asparagus ( Figure 2 ). This curve shows the probability that a fertile egg of *H. armigera* will survive to age *x* and stage *j* . This *sxj* curve can be simplified to the age-specific survival rate ( *lx* ) curve ( Figure 3 ) by ignoring the stage differentiation; the curve can then be calculated using Equation 4, including all individuals in the cohort. The female age-specific fecundity ( *fx* 10) (the female in the 10 ^th^ life stage), the age-specific fecundity ( *mx* ), the age-specific maternity ( *lxmx* ), and the cumulative reproductive rate

$(R_x = \sum_{y=0}^{x}l_{y}m_{y})$

of *H. armigera* are also plotted in Figure 3 . The curve of *fx* 10 shows the mean number of fertile eggs produced by a female adult at age *x* , while the curve of *mx* includes all individuals of age *x* .

**Figure 4.**
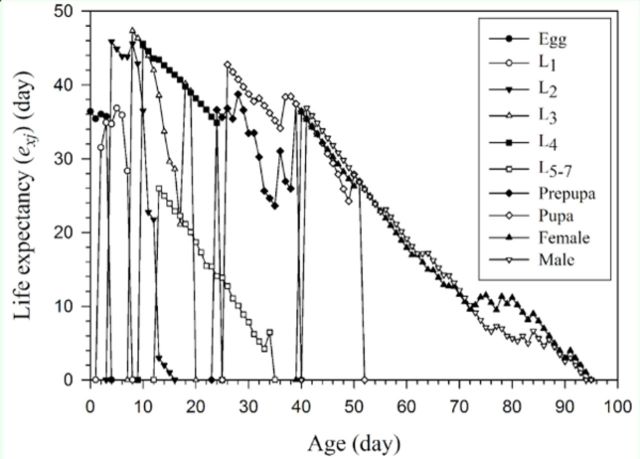
Age-stage-specific life expectancy ( *exj* ) of *Helicoverpa armigera* reared on asparagus. High quality figures are available online.

**Figure 5.**
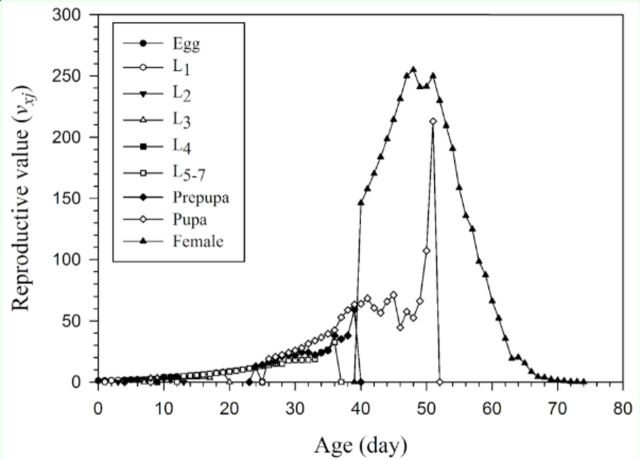
Age-stage-specific reproductive values ( *vxj* ) of *Helicoverpaarmigera* reared on asparagus. High quality figures are available online.

The age-stage life expectancy ( *exj* ) shows the total time that an individual of age *x* and stage *j* is expected to live ( Figure 4 ). Life expectancy shows a gradual decrease with aging be-because the laboratory has none of the adverse conditions observed in the field. The reproductive value ( *vxj* ) ( Figure 5 ) is defined as the contribution of an individual of age *x* and stage *j* to the future population ( Fisher 1930 ).

The mean consumption rate of each stage of *H. armigera* reared on asparagus is given in Table 3 . The mean consumption of total larval stage of those individuals that successfully developed to the adult stage was 2208.7 mg/individual, which was significantly higher than the mean consumption of the cohort (1183.02 mg/individual) due to the high immature mortality. The mean consumption rate of *H. armigera* larvae developing into males was, however, not significantly different from that of larvae developing into females ( Table 3 ).

**Table 3.**
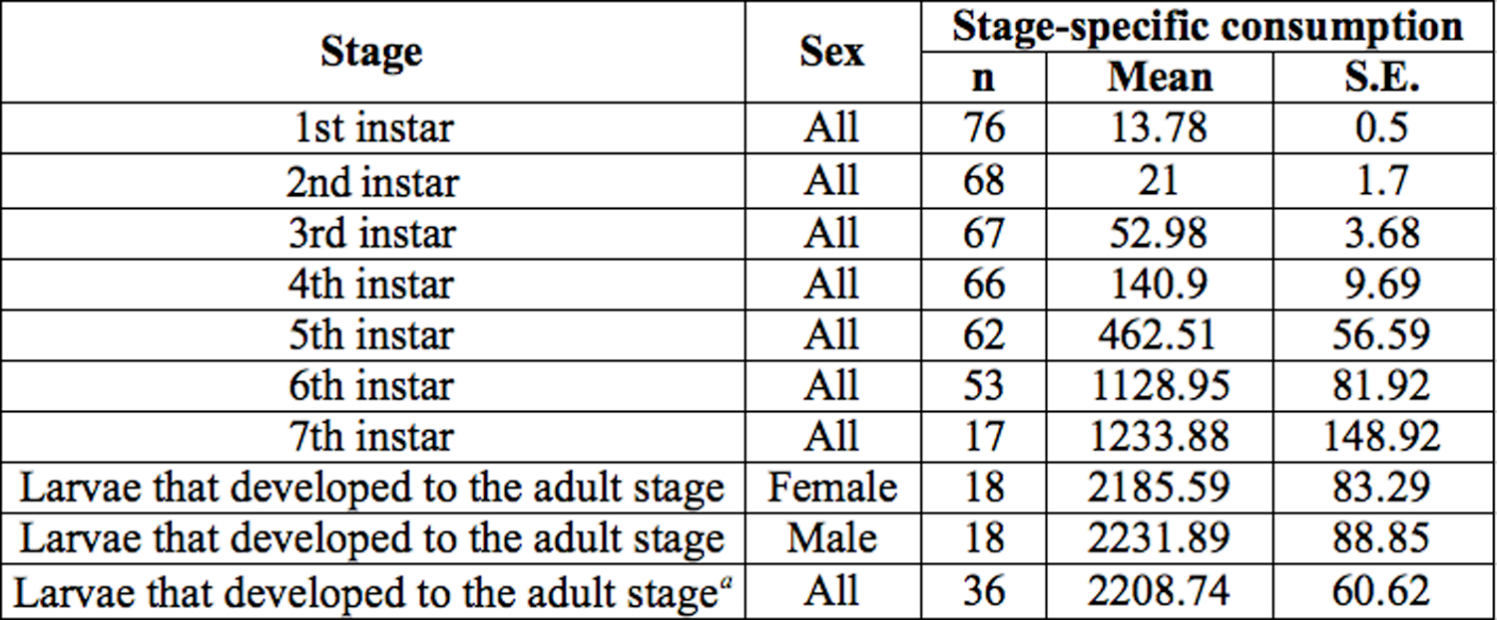
Stage-specific consumption (mg) of *Helicoverpa armigera* larvae reared on asparagus.

*^a^* This consumption rate includes all larvae that survived to the adult stage.

**Figure 6.**
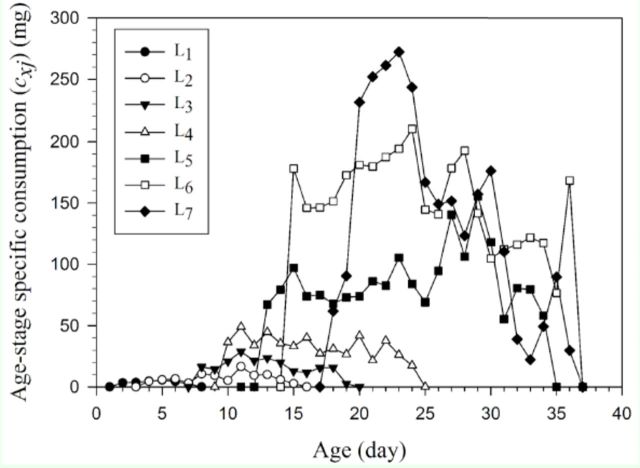
Age-stage-specific consumption rate ( *cxj* ) of *Helicoverpa armigera* reared on asparagus. High quality figures are available online.

The trends in the age-stage-specific consumption rate ( *cxj* ), i.e., the average amount of asparagus consumed by an individual of age *x* and stage *j* , are illustrated in Figure 6 . Along with the age-specific consumption rate ( *kx* ) and the age-specific net consumption rate ( *qx* ), the cumulative net consumption rate

**Figure 7.**
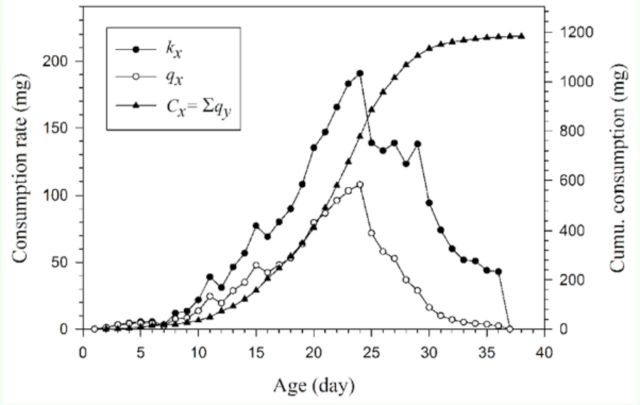
Age-specific consumption rate ( *kx* ), age-specific net consumption rate ( *qx* ), and cumulative consumption rate ( *Cx* ) of *Helicoverpa armigera* reared on asparagus. High quality figures are available online.

$(C_x = \sum_{y = 0}^{x}l_{y}k_{y} = \sum_{y = 0}^{x}q_y)$

of *H. armigera* was plotted in Figure 7 . The transformation rate ( *Qc* ), as calculated using Equation 6, was 17.48 mg/offspring, which is the amount of asparagus *H. armigera* must consume to produce one offspring (hatched egg).

**Figure 8.**
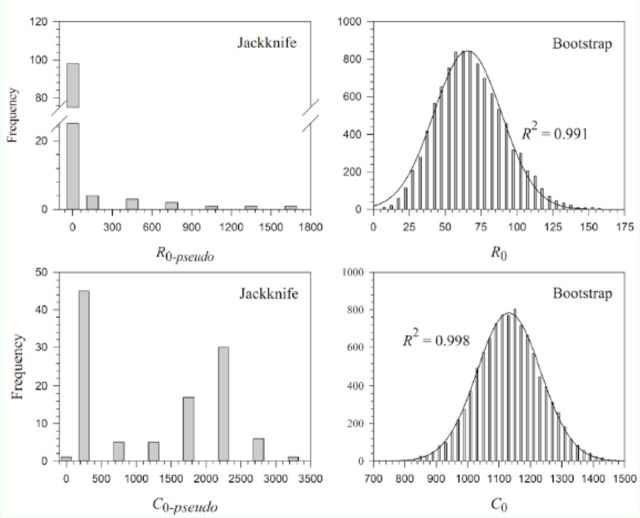
Frequency distribution of pseudovalues estimated by the jackknife technique and sample means estimated by the bootstrap technique (10,000 bootstraps) of the net consumption rate and the net reproductive rate of *Helicoverpa armigera* . High quality figures are available online.

The means and standard errors of *r, λ, R* 0, *GRR* , and *T* estimated by using the jackknife method and bootstrap method are listed and compared in Table 4 . Except *R* 0 in jackknife estimation, all population parameters of *H. armigera* reared on asparagus and reared on hybrid sweet corn were significantly different. In both estimations, the intrinsic rate ( *r* ) and the finite rate ( *λ* ) of *H. armigera* reared on asparagus were significantly lower than those reared on hybrid sweet corn. However, *T* and *GRR* for *H. armigera* reared on asparagus were significantly higher. When only the means and standard errors were concerned, there were only minor differences between the results estimated by these two techniques; however, the frequency distribution of estimated means (pseudo values) by the jackknife technique failed the normality test, while the frequency distribution of sample means of 10,000 bootstraps fitted the normal distribution well ( Figure 8 ). Moreover, the significant difference in *R* 0 of *H. armigera* reared on asparagus and hybrid sweet corn were not detected by using jackknife techniques ( Table 2 ).

**Table 4.**
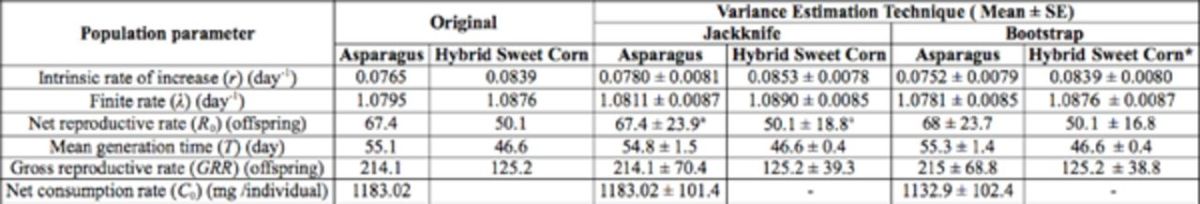
Comparison of population parameters of *Helicoverpa armigera* reared on asparagus and hybrid sweet corn estimated by the jackknife and bootstrap methods.

^*^ by reanalyzing the data used in Jha et al. (2012)

Sample size of asparagus ( *n* = 110) and hybrid sweet corn ( *n* = 98) for original and jackknife estimation. Bootstrap size for both asparagus and hybrid sweet corn was 10000. The means of population parameters of asparagus and hybrid sweet corn under jackknife or bootstraps were significantly different ( *P* < 0.001) using the U-test, except those followed by the same letter.

## Discussion

This study comprehensively presents the life history and demography of *H. armigera* reared on asparagus. Asparagus is recorded as a host of *H. punctigra* in Australia (in Table 1 of Zalucki et al. 1986 ). The completion of the life cycle on asparagus foliage and the production of fertile offspring by *H. armigera* in the laboratory during this study, as well as the occurrence of this insect in asparagus fields, in-indicates that asparagus is a natural host for *H. armigera* ( de Boer and Hanson 1984 ; COA 1996 ; Bussell et al. 2002 ; Fei et al. 2010 ). As a polyphagous pest, *H. armigera* may occur simultaneously on several hosts within a region and persist throughout the host’s growing season. Fitt (1989) reported that insects of *Heliothis* spp. can persist at low density in a seemingly unsuitable host or area. Thus, asparagus may play a role in supporting the build-up of large populations of *H. armigera* in diversified crop areas where it is introduced and cultivated in vicinity to more suitable hosts such as sweet corn.

The larval duration (25.33 days) and the total duration of immaturity (45.33 days) of *H. armigera* reared on asparagus observed in this study were longer than those observed in larvae raised on hybrid sweet corn ( Jha et al. 2012 ) and other host plants, such as eggplant, pepper, okra, and tomato ( Jallow et al. 2001 ) *.* Seven larval instars of *H. armigera* were observed during rearing on asparagus, whereas only six larval instars were observed on the artificial diet and hybrid sweet corn ( Jha et al. 2012 ). The duration of the pupal stage (14.19 days) in *H. armigera* reared on asparagus was similar to that reported in pupae raised on hybrid sweet corn and the other host plants mentioned above. This result agrees with the earlier finding that there was no apparent effect of larval food and larval growth rate on duration of the pupal phase of *H. armigera* ( Twin 1978 ); however, the pupa reared on asparagus weighed the least of the reported pupal weights of this insect fed with the host plants mentioned above and significantly lighter than those on hybrid sweet corn. The maximal longevity on asparagus foliage was 95 days, which, to date, is the longest recorded time period needed by *H. armigera* to develop on different host plants under varying conditions. When the larvae were reared on asparagus, the rate of survival to the adult stage was 32.73%, which was considerably less than the rate observed in the cohort reared on hybrid sweet corn (46.94%) and artificial diet (56.60%) ( Jha et al. 2012 ). This difference is consistent with the finding of Jallow et al. (2001) that the type of food consumed by larvae influenced the survival rate to adult. Development tends to be slower in individuals reared on plant materials than in those fed artificial diets ( Zalucki et al. 1986 ). Moreover, nitrogen and total nonstructural carbohydrate content in asparagus foliage is lower ( Table 5 ) than those reported for the leaves of other vegetables, e.g., cabbage ( Hsu et al. 2009 ) and radish ( Yadav et al. 2010 ). This indicates poor nutritional quality of asparagus foliage that may be a cause for the poor performance of *H. armigera* in it.

**Table 5.**
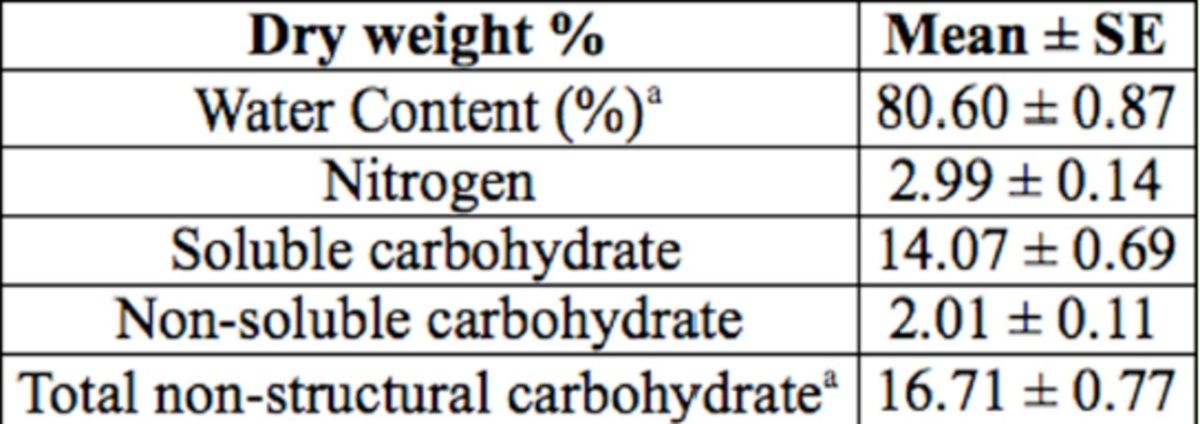
Water content, nitrogen, and total nonstructural carbohydrate concentration in asparagus foliage (n = 9).

^a^ Total nonstructural carbohydrate = soluble carbohydrate + non-soluble carbohydrate

The variation in survival rate, developmental rate, and fecundity among *H. armigera* individuals can be observed in the overlapping *sxj* curves ( Figure 2 ), the *exj* curves ( Figure 4 ), and the *vxj* curves ( Figure 5 ). These curves clearly illustrate stage differentiation in an *H. armigera* cohort. Individuals in the cohort completed their larval stage at the 5 ^th^ , 6 ^th^ , and 7 ^th^ instars. Among those individuals that developed to the adult stage from the 6 ^th^ instar, the duration of the 4 ^th^ , 5 ^th^ , and 6 ^th^ instars and the total duration of the larval stage differed significantly from those observed in individuals that developed to the adult stage through the 7 ^th^ instar ( Table 2 ). The duration of the pupal phase in males (15.06 days) was significantly different from the duration in females (13.33 days) ( *P* < 0.001). Additionally, variations among individuals influenced the population characteristics of *H. armigera* . The age-stage fecundity of *H. armigera* , calculated by the number of eggs hatched, produces more realistic estimates rather than models based on the total number of eggs laid because the hatch rate varies with the mother’s age ( Jha et al. 2012 ). In this study, all of these variations were duly considered.

In comparison to hybrid sweet corn, the lower *r* and *λ* , longer *T* , more larval instars, and longer stage-specific preadult duration revealed the poor fitness of *H. armigera* on asparagus. The percentage of oviposition days was 29.96% on hybrid sweet corn and 34.26% on asparagus. This may be a reason for higher *R* 0 and *GRR* of *H. armigera* on asparagus. Liu et al. (2004) used *R* 0 for evaluating the suitability of host plants. However, *R* 0 and *GRR* only represent the reproductive potential rather than the overall fitness. Thus, *R* 0 should be used cautiously while evaluating suitability of host plants to an insect. Based on the above discussion, asparagus foliage can be viewed as a less suitable host for *H. armigera* in comparison to hybrid sweet corn. This may be due to nutritional inferiority of vegetative tissues and poor palatability of asparagus foliage. Additional studies are needed for a comprehensive comparison.

The results of this study fully illustrate the concept of the age-stage, twosex life table and elucidate stage differentiation during the growth and development of *H. armigera* . The inevitable problems in the traditional female age-specific life table ( Lewis 1942 ; Leslie 1945 ; Birch 1948 ; Caswell 1989 ; Carey 1993 ) and the problems of calculating *lx* and *mx* based on adult age were discussed in detail in Chi (1988) , Yu et al. (2005) , Chi and Su (2006) , Kavousi et al. (2009) , and Huang and Chi (2012a) with mathematical proofs. The results of this study are consistent with the relationship between *R* 0 and *GRR* ( Yu et al. 2005 ) as well as the relationship between the *F* and *R0* ( Chi 1988 ; Chi and Su 2006 ). In this study, the age-stage consumption rate was integrated into the age-stage, twosex life table. This integration enabled us to simultaneously study the population-level consumption and the life table from the same cohort of *H. armigera* and facilitated the estimation of the net consumption rate and intrinsic rate of increase. The intrinsic rate of increase is considered the most appropriate measure of fitness ( Smith 1991 ). Similarly, the net consumption rate represents the consumption capacity of a pest population, including all individuals of both sexes and those that died before reaching the adult stage ( Chi and Yang 2003 ; Yu et al. 2005 ; Chi and Su 2006 ; Farhadi et al. 2011).

Consumption is the primary component of the nutritional ecology of insects ( Scriber and Slansky 1981 ; Greenberg et al. 2001 ), is a key variable in establishing a link between pest injury and crop loss, and is thus of great importance in modeling the economic injury level and the economic threshold for integrated pest management ( Pedigo et al. 1986 ; Lee et al. 2011 ). The consumption rate of *H. armigera* was determined to assess its performance on different host plants or diets ( Singh 1999 ; Ahmad 2002 ; Hamed and Nadeem 2008 ; Lin et al. 2008 ; Bhonwong et al. 2009 ; Gopala et al. 2010 ; Naseri et al. 2011 ) and to compare the population fitness of this species across varying climatic conditions ( Wu et al. 2006 ; Yin et al. 2009 , 2010); however, the published information on consumption patterns in *H. armigera* includes only stage-specific consumption information. Because the consumption rate of *H. armigera* varies with age and stage, consumption rates based on stage-specific consumption are insufficient for developing a pest-management program based on life-table data. Nibouch et al. (2007) used the cumulative consumption by all larval stages to model damage caused by *H. armigera* . Larval consumption calculated by summing the stage-specific consumption of each instar and ignoring the stage-specific mortality (Table 1 of Nibouch et al. 2007) will yield an overestimate at the population level. The net consumption rate estimated, in contrast, is a more precise value that is suitable for use in modeling because it considers age-stage survival in calculating consumption.

In this paper, the jackknife and bootstrap resampling methods were compared. The bootstrap method generated a normal distribution of estimated means, which facilitates further robust statistical examination. Chi (1988) , Yu et al. (2005) , and Chi and Su (2006) provided mathematical proofs of the relationships among mean fecundity ( *F* ), net reproductive rate ( *R* 0), and gross reproductive rate ( *GRR* ). The estimated values from both the jackknife and bootstrap methods, however, showed slight inconsistencies in these relationships. Moreover, application of jackknife technique to *R* 0 was mathematically invalidat-ed by Huang and Chi (2012b) , who suggested not to use it for the estimation of variability of *R* 0. Therefore, mathematical validation for the choice of resampling techniques for other parameters needs to be studied.

Age-stage, twosex life table theory helps construct a comprehensive life table describing the demographic characteristics of insect and mite populations ( Chi and Su 2006 ). This tool allows the description of the stage differentiation of *H. armigera* and the incorporation of this parameter into precise estimations of derived population parameters ( Chi and Liu 1985 ).

A correct understanding of a pest’s life table is essential for implementing an ecology-oriented management program ( Zalucki et al. 1986 ). By integrating studies on consumption rate into life-table studies and by considering variations due to age, stage, and sex, the species’ growth, stage differentiation, reproduction, and consumption rate can be effectively characterized ( Chi and Yang 2003 ). Thus, we recommend that the age-stage, twosex life table be used in insect demographic studies to obtain accurate basic and derived population parameters for population growth projections, for designing mass rearing programs, for pest management, and for studies of insect ecology.
